# Phenotypic and genotypic characterization of multidrug resistant and extended-spectrum β-lactamase-producing *Enterobacterales* isolated from clinical samples in the western region in Cameroon

**DOI:** 10.1186/s12879-023-08742-7

**Published:** 2023-11-22

**Authors:** Omer Aurelle Nkengkana, Raspail Carrel Founou, Luria Leslie Founou, Brice Davy Dimani, Patrice Landry Koudoum, Jessica Ravalona Zemtsa, Aurelia Mbossi, Cyrielle Signe Mawout, Larissa Tchakounte Tegang, Michel Noubom

**Affiliations:** 1 Department of Microbiology- Hematology and Immunology, Faculty of Medicine and Pharmaceutical Sciences, University of Dschang, Dschang, Cameroon; 2 Antimicrobial Research Unit, School of Health Sciences, College of Health Sciences, University of KwaZulu-Natal, Durban, 4000 South Africa; 3 Antimicrobial Resistance and Infectious Disease (ARID) Research Unit, Research Institute of Centre of Expertise and Biological Diagnostic of Cameroon (CEDBCAM-RI), Yaoundé, Cameroon; 4 Reproductive, Maternal, Newborn and Child Health (ReMARCH) Research Unit, Research Institute of the Centre of Expertise and Biological Diagnostic of Cameroon (CEDBCAM-RI), Yaoundé, Cameroon; 5 Bioinformatics & Applied Machine Learning Research Unit, EDEN Biosciences Research Institute (EBRI), EDEN Foundation, Yaoundé, Cameroon; 6 Annex Regional Hospital of Dschang (ARHD), Dschang, Cameroon

**Keywords:** Antibiotic resistance, *Enterobacterales*, ESBLs, CTX-M, Cameroon

## Abstract

**Background:**

The 2017 World Health Organization (WHO) report has listed extended-spectrum β-lactamase-producing *Enterobacterales* (ESBL-E) as critical pathogens for public health and requiring urgently new antibiotics. The aim of this study was to characterize phenotypically and genotypically ESBL-E isolated among clinical samples in Dschang, Cameroon.

**Methods:**

A cross-sectional study was conducted during a four-month periods from February to May 2022 in the two biggest hospitals of Dschang. Clinical samples were collected and cultured on Eosin Methylene Blue agar. Suspected growing colonies were biochemically identified using the Enterosystem Kit 18R. Antimicrobial susceptibility testing (AST) was done using the Kirby Bauer disc diffusion method and interpretated according to the CA-SFM recommendations. ESBL phenotypes were double screened using CHROMagar™ ESBL and double disk synergy test (DDST). The detection of resistance genes was performed using conventional and multiplex PCR methods. Results were analyzed with SPSS (version 21) and a p-value < 0.05 was considered statistically significant.

**Results:**

A total of 152 *Enterobacterales* were isolated among 597 clinical samples including urine, blood, cervico-vaginal, urethral swabs and wound samples. The overall prevalence of ESBL-*Enterobacterales* was 29.61% (45/152). The most represented ESBL species were *Escherichia coli* (n = 23; 51.11%), *Klebsiella pneumoniae* (n = 8; 17.78%) and *Citrobacter freundii* (n = 6; 13.33%).

**Conclusion:**

This study reveals the high burden of ESBL-E among clinical samples in the regional hospital in Dschang with the most common species being *E. coli* and *K. pneumoniae*. It confirmed the high occurrence of *bla*_CTX−M_ and *bla*_TEM_ among ESBL-E. The study suggests that implementing antimicrobial stewardship program and real-time surveillance of antimicrobial resistance are needed in the Western region of Cameroon. Moreover, the implementation of infection prevention and control measures (IPC) is essential to curb the dissemination of these bacteria from community to hospital settings. Implementation of national action plan to fight against antimicrobial resistance at the local levels is urgently needed.

## Background

Extended-spectrum β-lactamase (ESBL) and carbapenemase-producing *Enterobacterales* have been listed by the World Health Organization (WHO) as critical priority pathogens for which new antimicrobials need to be developed [1]. To escape the activities of the majority of β-lactam antibiotics such as penicillin, first, second and third generations of cephalosporin (C3G), *Enterobacterales* produced ESBL enzymes leading to increase resistance to all β-lactam except cephamycins, carbapenems and monobactams [2]. *Escherichia coli* and *Klebsiella pneumoniae* are among the leading species associated with ESBL production [3]. They are responsible for the increased hospitalization length of stay and deaths particularly in Low-and-Middle Income Countries (LMICs) [3–8].

Multi-drug resistance is increasingly being detected in numerous clinical *Enterobacterales* strains because of the extensive antibiotic used in hospital settings [3]. Despite considerable efforts for their containment, ESBL-producing *Enterobacterales* (ESBL-E) and multi-drug resistant *Enterobacterales* (MDR-E) are increasingly implicated in several difficult-to-treat infections especially in sub-Saharan African (sSA) countries [3].

Numerous genes encoding for ESBL enzymes were implicated and described among clinical isolates in Uganda, Nepal, Côte d’Ivoire and Chad with the most important genes being *bla*_CTX−M_ followed by *bla*_TEM_ and *bla*_SHV_ [8–11]. Moreover, a high prevalence of ESBL-E were also observed from carriage among HIV patients during a COVID-19 pick in 2022 in Cameroon [12].

ESBL enzymes have emerged following chromosomal mutation and acquisition of resistance genes carried on diverse mobile genetic elements (MGEs) including plasmids, integrons, insertion sequences, transposons, genomic islands and bacteriophage [11]. The common transferability of resistance amongst bacteria will likely be associated with increasing rates of MDR infections, although some gaps remain as the burden of MDR-E in community as well as hospitalized patients [11]. This study aims at determining the phenotypic and genotypic characteristics of ESBL-E isolated from clinical samples in two hospitals in Dschang, Cameroon post COVID-19 pandemic.

## Methods

### Study settings

This study was carried out in the two biggest health care structures of the Dschang District in Western region of Cameroon encoded as H1 and H2 for ethical reasons. The H1 is the biggest health care infrastructure in Dschang while H2 is a private Catholic Medical Center with the same level. Clinical samples were collected and analyzed during a four-month periods, from February 1st to May 20th, 2022. All clinical samples originating from community and hospitalized patients were examined in the respective laboratories of these hospitals.

### Samples collection and identification

All collected samples except feces were inoculated onto Eosin Methylene Blue (EMB) agar (CM-EMB100, Rapid Labs). All fecal samples were inoculated into Selenite broth and incubated for 18 to 24 h at 37 °C. After incubation, the inoculum was plated onto and Salmonella-Shigella (SS) agar (CM-SSB244, Rapid Labs) and was then incubated for 18 to 24 h at 37 °C for the isolation of *Salmonella spp.* as well as *Shigella spp*. For all growing colonies, oxidase test was performed to identify Gram negative fermenting bacteria. Specie identification was performed using the Enterosystem 18R kits following the manufacturer’s recommendations.

### Antimicrobial susceptibility testing (AST) and ESBL screening methods

The AST was performed using the Kirby-Bauer disc diffusion method on Muller Hinton agar following the recommendations of the Antibiogram Committee of the French Society of Microbiology (CA-SFM) guidelines. A panel of 11 antibiotic discs of five different families were tested including: amoxicillin and clavulanic acid (AMC; 20 − 10 µg), ceftazidime (CAZ; 30 µg), cefotaxime (CTX; 30 µg), ceftriaxone (CRO; 30 µg), cefixime (CXM; 30 µg), imipenem (IMP; 10 µg), gentamicin (CN; 30 µg), tobramycin (TOB; 10 µg), ciprofloxacin (CIP; 5 µg), levofloxacin (LEV; 5 µg) and chloramphenicol (CHL; 30 µg). A double ESBL-E screening was done using double-disk synergy method and confirmed using ESBL chromogenic agar CHROMagar ESBL (CHROMagar™ Orientation, Paris - France). The samples were then stored at -20 °C in cryotubes containing trypticase soya supplemented with 20% glycerol.

### DNA genomic extraction

Extraction of the genomic DNA of all ESBL-E isolates subjected to molecular characterization was done as previously described [12]. During the procedure, two pure colonies of ESBL-E were introduced into 400 µL of a solution of Tris EDTA (10 mM Tris; 0.1 mM EDTA), then the suspension was boiled at 95 °C for 25 min using a dry bath (MIULab DKT200-1, Lasec International Ltd., Johannesburg, South Africa). This was followed by centrifugation of the suspension at 9500 rpm for 5 min. Finally, 150 µL of the supernatant containing the DNA was transferred to an eppendorf tube and frozen at -20 °C for further analysis.

### Detection of ESBL-producing ***Enterobacterales***

#### Conventional and multiplex PCR methods

A 10 µL PCR master mix solution was used for detection of the *bla*_SHV_ gene among ESBL-E isolates and consisted of 2.8 µl of nuclease-free water, 0.1 µl of each forward and reverse primer, 5 µl of 2x DreamTaq green PCR master mix (Thermo Fisher Scientific, Lithuania) and 2 µl of DNA. Singleplex PCR was performed using BIO-RAD T100 thermal cycler (Bio-Rad Laboratories, Marnes-la-Coquette, France). The amplification steps were as follows: initial denaturation (94 °C for 30 s), 30 cycles of denaturation at 94 °C for 4 s, elongation at 72 °C for 50 s and final elongation at 72 °C for 5 min.

For the detection of the *bla*_TEM_ and *bla*_CTX−M_ genes among the ESBL-E, the reaction occurred in a 10 µl reaction mix with a volume consisting of 5 µL of 2x DreamTaq green PCR Master mix (Thermo Fisher Scientific, Lithuania), 2.6 µL of nuclease-free water, 0.1 µL of each reverse and forward primer and 2 µL of template DNA. The amplification steps for all reactions were as follows: initial denaturation (94 °C for 30 s), 30 cycles of denaturation at 94 °C for 4 s, annealing for 40 s, elongation at 72 °C for 50 s and final elongation at 72 °C for 5 min [13, 14].

### PCR products and visualization

Revelation of amplicons was done along with a 100 bp molecular ladder (New England Biolabs, MA, USA) in a 1.5% (w/v) agarose gel electrophoresis. Migration was set at 90 V for 45 min followed by staining with ethidium bromide solution (0.5 µg/mL) for 15 min and rapid destaining in water. The visualization was made under ultraviolet radiation using a G-BOX chemi XL gel documentation system (Syngene, Cambridge, UK).

### Data analysis

Socio-demographic and clinical data were entered into Epi Info version 7.2.5.0 and exported to SPSSv21 for analysis. Pearson Chi-square test was used to assess for any differences between the two ESBL phenotype categories with respect to clinical and demographic parameters. The different data, were compared using the independent t-test. A p-value < 0.05 was considered statistically significant.

## Results

### Socio-demographic characteristics of patients

**Fig. 1:**
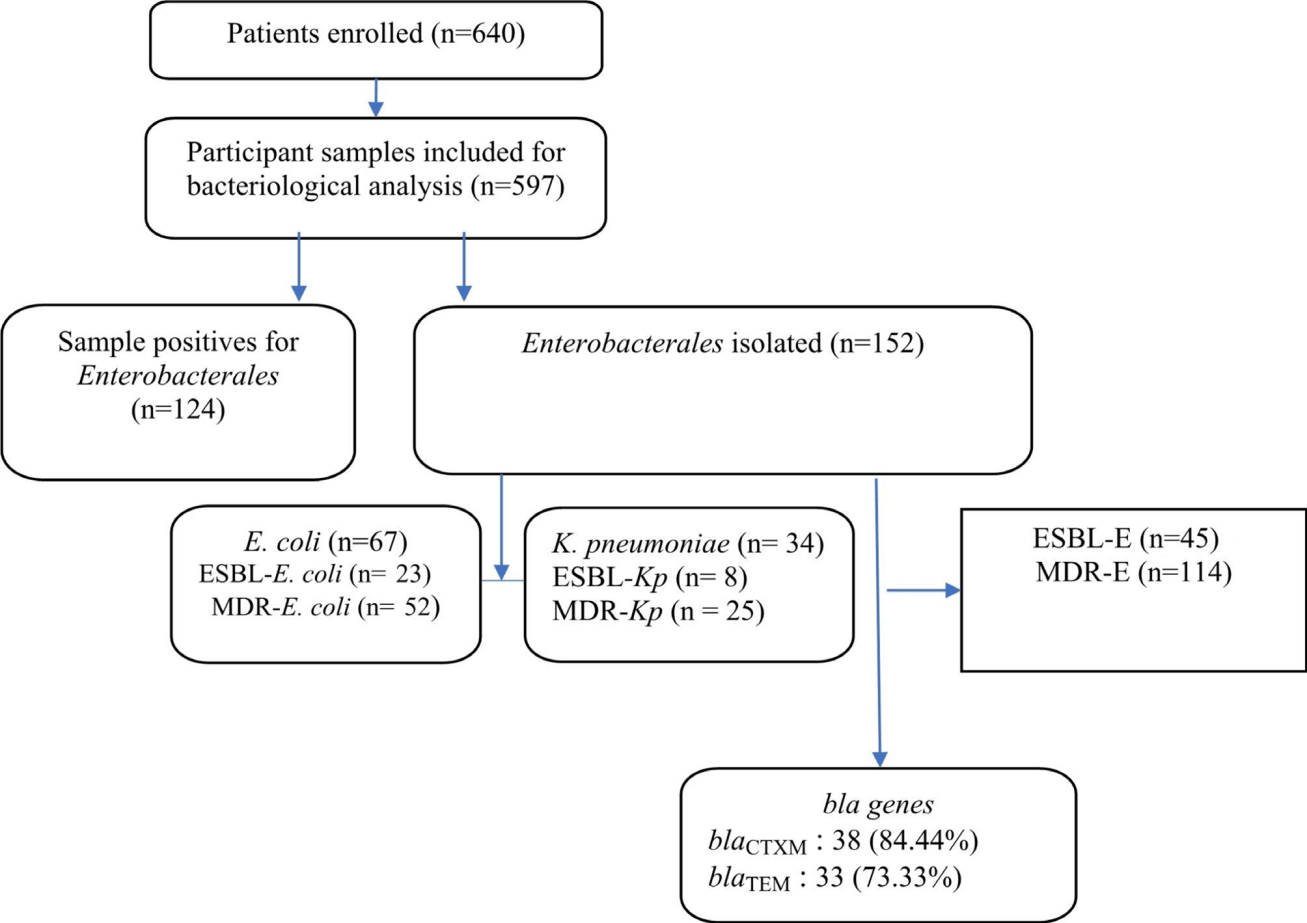
Flowchart of participants and isolates

A total of 597 consecutive clinical samples from patients attended to hospitals was collected from the laboratories of both hospitals H1 and H2 while 124 samples were positive with at least one *Enterobacterales* with a total of 154 *Enterobacterales* identified (Fig. 1). Most of the study participants were females, 83.25% (497/597), whereas the age group 20–30 years old (n = 280/597; 46.90%) was most frequent. Overall, 20.77% (n = 124/597) of participants had a culture positive to *Enterobacterales*, with 29.84% (n = 37/124) of these being infected by an ESBL-E isolates and 76.61% (n = 95/124) by MDR-E. The majority of patients infected by *Enterobacterales* were living in urban area (n = 101/124; 81.45%) comparing to rural area (18/124; 14.52%) with a significant difference (P < 0.0001).

A total of 75.88% (453/597) and 24.12% (144/597) samples were collected in H1 and H2, respectively. We observed 63.71% (79/124) and 36.29% (45/124) samples positives to *Enterobacterales* culture in H1 and H2, respectively. The 63.2% (60/95) and 36.8% (35/95) of patients infected by MDR-*Enterobacterales* were coming from H1 and H2, respectively with a significant difference (P < 0.05). Concerning ESBL-E, 70.3% (26/37) and 29.7% (11/37) of patients infected were coming from H1 and H2, respectively. The age of the participants was between 1 and 95 years as illustrated in Table 1.

**Table 1:** sociodemographic characteristics of participants

Variables	Modalities	Overall		*Enterobacterales* positive		ESBL-PE		MDR-E	
		N (%)	Percentage (%)	N (%)	*p-*value	N (%)	*p-*value	N (%)	*p-*value
Overall		597		124		37		95	
Sex Sex ratio : 1.2013	Female	497	83.25	99 (79.84)	0.2532	27 (73.0)	0.2135	76 (80.0)	0.4597
	Male	100	16.75	25 (20.16)		10 (27.0)		19 (20.0)	
Age	Mean (SD)			34.3 (16.6)	**0.0010**	37.5 (16.8)	**0.0021**	35.2 (16.2)	**0.0013**
	Median (Min, Max)			30.0 [6.00, 92.0]		34.0 [7.0,70.0]		30.0 [6.0,92.0]	
Age group	]1 ; 10]	25	4,19	7 (5.65)	**0.0009**	1 (4.00)	**0.0033**	4 (4.21)	**0.0042**
	]10 ; 20 ]	68	11,39	9 (7.26)		2 (2.94)		6 (6.32)	
	]20 ; 30]	280	46,90	49 (39.52)		15 (5.36)		39 (41.05)	
	]30 ; 40]	122	20,44	23 (18.55)		4 (3.28)		17 (17.89)	
	]40 ; 50]	45	7,54	16 (12.90)		7 (15.56)		12 (12.63)	
	]50 ; 60]	21	3,52	11 (8.87)		4 (19.05)		10 (10.53)	
	> 60	30	5,03	9 (7.26)		4 (13.33)		7 (7.37)	
	≤ 1	6	1,01	0 (0)		0 (0)		0	
Residence	Dschang rural area	347	58.12	18 (14.52)	**< 0.0001**	10 (27.0)		15 (15.8)	**< 0.0001**
	Dschang urban area	222	37.19	101 (81.45)		24 (64.9)		76 (80.0)	
	Other#	28	4.69	5 (4.03)		3 (8.1)		4 (4.2)	
Clinical samples	Blood	19	3,18	2 (1.61)	**< 0.0001**	1 (5.26)		2 (2.1)	**< 0.0001**
	Cerebrospinal fluid	10	1,68	0 (0)		0 (0)		0	
	Other puncture fluid	11	1,84	0 (0)		0 (0)		0	
	Semen	10	1,68	1 (0.81)		0 (0)	**< 0.0001**	1 (1.1)	
	Stool	81	13,57	46 (37.10)		16 (19.75)		30 (31.6)	
	Urine	55	9,21	10 (8.06)		3 (5.45)		8 (8.4)	
	Uro-genital swab	396	66,33	63 (50.81)		15 (3.79)		52 (54.7)	
	Wound	16	2,68	2 (1.61)		0		2 (2.1)	
Occupation	Chef	1	0,17	1 (0.81)	0.0679	0	0.2041	1 (1.1)	0.1963
	Civil engineering	6	1,01	2 (1.61)		0		2 (2.1)	
	Dressmaker	31	5,19	7 (5.65)		0		6 (6.3)	
	Driver	6	1,01	0 (0)		0		0 (0.0)	
	Farmer	14	2,35	7 (5.65)		2 (14.29)		6 (6.3)	
	Hairdresser	15	2,51	1 (0.81)		0 (0)		1 (1.1)	
	Healthworker	12	2,01	4 (3.23)		1 (8.33)		3 (3.1)	
	Housemaid	156	26,13	35 (28.23)		15 (9.62)		25 (26.3)	
	Merchant	46	7,71	13 (10.48)		5 (10.87)		9 (9.5)	
	‘NA’	11	1,84	1 (0.81)		0 (0)		1 (1.1)	
	Other	9	1,51	2 (1.61)		0 (0)		2 (2.1)	
	Pupil	53	8,88	11 (8.87)		1 (1.89)		6 (6.3)	
	Secretariat	5	0,84	0 (0)		0 (0)		0 (0.0)	
	Student	165	27,64	25 (20.16)		7 (4.24)		19 (20.0)	
	Teacher	67	11,22	15 (12.10)		5 (7.46)		14 (14.7)	
Sanitary facilities	H1	453	75.88	79 (63.7)	**0.0004**	26 (70.3)	**0.0010**	60 (63.2)	**0.0017**
	H2	144	24.12	45 (36.3)		11 (29.7)		35 (36.8)	
*Enterobacterales* positive	No	473	79.23		/	0	**< 0.0001**	0	**< 0.0001**
	Yes	124	20.77			37 (100)		95 (100)	
ESBL positives	No	552	92.46	87 (70.2)	**< 0.0001**		/	65 (68.4)	**< 0.0001**
	Yes	45	7.54	37 (29.8)				30 (31.6)	
MDR	No	483	80.90	29 (23.4)	**< 0.0001**	7 (18.9)	/		
	Yes	114	19.10	95 (76.6)		30 (81.1)			

**#Other including Bafoussam, Penka Michel, Santchou, Douala, Yaoundé**

**Other puncture fluids including ascit fluid, breast fluid, pleural liquid**

### Frequency of ***Enterobacterales*** isolates

Out of these 597 samples analyzed, 360 (60,30%) were culture positive to bacteria of which 124 (20.77%) were positive to one or more *Enterobacterales*. Out of the *Enterobacterales* positive samples, 152 *Enterobacterales* isolates were identified. *Escherichia coli* (n = 67; 44.07%), *Klebsiella pneumoniae* (n = 34; 22.36%), *Citrobacter freundii* (n = 16; 10.52%) and *Serratia liquefaciens* (n = 10; 06.57%) were the leading species. The isolates were identified from a various clinical specimens collected: endocervical swabs (n = 75; 49.34%), stool (n = 61; 40.13%) and other samples (n = 16; 10.52%) as illustrated in Table 2. *E. coli* was mostly detected among endocervical swabs (n = 41/67; 61.19%) and stool (n = 19/67; 28.36%) followed by *K. pneumoniae* among vaginal swabs (n = 20/34; 58.82%) and stool (n = 8/34; 23.53%) respectively, while *C. freundii* was only isolated from stool (n = 14/14; 100%). Among all identified *Enterobacterales*, the majority (122/152; 80.26%) were coming from patients living in urban area. Concerning hospitals, majority of isolates were coming from H1 (93/152; 61.18%) and (59/152; 38.22%) from H2 as illustrated in Table 2.

**Table 2:** Distribution of *Enterobacterales* Species according to socio-demographic characteristics. collection sites and types of samples

Variables	Modalities	Isolates MDR Positive	Isolates ESBL-PE		*Enterobacterales* Species	*E. coli*	*K. pneumoniae*	*C. freundii*	*S. liquefaciens*	*Salmonella spp*	*Others isolates*	*p-value*
Overall		**114**	45		152	67	34	14	10	8	19	
Sex	Female	**90 (78.95)**	35 (77.78)		120 (78.95)	56 (46.67)	29 (24.17)	8 (6.67)	9 (7.50)	5 (4.17)	13 (10.83)	0.042*
	Male	24 (21.05)	10 (22.22)		32 (21.05)	11 (34.38)	5 (15.63)	6 (18.75)	1 (3.13)	3 (9.38)	6 (18.75)	
Age groups	]0 ; 10]	5 (4.39)	01 (2.22)		9 (5.92)	3 (33.33)	1 (11.11)	1 (11.11)	0	2 (22.22)	2 (22.22)	0.729
	]10 ; 20]	8 (7.02)	2 (4.44)		12 (7.89)	5 (41.67)	1 (8.33)	1 (8.33)	2 (16.67)	2 (16.67)	1 (8.33)	
	]20 ; 30]	**46 (40.35)**	18 (40)		60 (39.47)	25 (41.67)	14 (23.33)	5 (8.33)	5 (8.33)	2 (3.33)	9 (15)	
	]30 ; 40]	18 (15.79)	04 (8.89)		24 (15.79)	11 (45.83)	6 (25)	2 (8.33)	2 (8.33)	1 (4.17)	2 (8.33)	
	]40 ; 50]	15 (13.16)	08 (17.78)		19 (12.50)	9 (47.37)	6 (31.58)	2 (10.53)	1 (5.26)	0	1 (5.26)	
	]50 ; 60]	11 (9.65)	06 (13.33)		14 (9.21)	9 (64.29)	3 (21.43)	1 (7.14)	0	0	1 (7.14)	
	> 60	11 (9.65)	06 (13.33)		14 (9.21)	5 (35.71)	3 (21.43)	2 (14.29)	0	1 (7.14)	3 (21.43)	
Residence	Dschang Urban area	**90 (78.95)**	28 (62.22)		122 (80.26)	51 (41.80)	26 (21.31)	12 (9.84)	9 (7.38)	7 (5.74)	17 (13.93)	0.999
	Dschang Rural area	19 (16.67)	13 (28.89)		23 (15.13)	11 (47.83)	6 (26.09)	2 (8.70)	1 (4.35)	1 (4.35)	2 (8.70)	
	Others	5 (4.39)	4 (8.89)		8 (5.26)	5 (71.43)	2 (28.57)	0	0	0	0	
Sanitary facilities	H1	**70 (61.40)**	32 (71.11)		93 (61.18)	47 (50.54)	13 (13.98)	7 (7.53)	5 (5.38)	6 (6.45)	15 (16.13)	0.181
	H2	44 (38.60)	13 (28.89)		59 (38.22)	20 (33.90)	21 (35.59)	7 (11.86)	5 (8.47)	2 (3.39)	4 (6.78)	
Samples	Stool	41 (35.96)	19 (42.22)		61 (40.13)	19 (31.15)	8 (13.11)	14 (22.95)	2 (3.28)	7 (11.48)	11 (18.03)	0.0001*
	Urine	8 (7.02)	03 (6.67)		10 (6.58)	5 (50)	4 (40)	0	1 (10)	0	0	
	Blood	2 (1.75)	01 (2.22)		2 (1.32)	1 (50)	1 (50)	0	0	0	0	
	Endocervical swab	**59 (51.75)**	20 (44.44)		75 (49.34)	41 (54.67)	20 (26.67)	0	7 (9.33)	1 (1.33)	6 (8)	
	Urethral swabs	1 (0.88)	0		1 (0.66)	1 (100)	0	0	0	0	0	
	Wounds	2 (1.75)	02 (4.44)		2 (1.32)	0	1 (50)	0	0	0	1 (50)	
	Semen	1 (0.88)	0		1 (0.66)	0	0	0	0	0	1 (100)	

*Statistically significant value

### Occurrence of ESBL-producing ***Enterobacterales***

Out of the 152 non-duplicated *Enterobacterales* identified, 45 (29.60%) were ESBL producers. The most common ESBL species were *E. coli* (51.11%; 23/45), *K. pneumoniae* (17.77; 8/45) and *C. freundii* (13.33%; 6/45) as described in the Table 3. The proportion of ESBL-E was higher in H1 (71.11%; 32/45) than H2 although without statistical significance (71.11% vs. 28.29%; 13/45) (p = 0.1811). ESBL-E were frequently isolated from vaginal swabs (44.44%; n = 20/45), fecal samples (42.22%; n = 19/45) and urine (6.67%; n = 3/45) (Table 2).

**Table 3:** Antibiotic resistance profiles of *Enterobacterales* isolated from clinical samples

Isolates (n)	Resistance rate to antibiotics, n(%)										
	**AMC**	**CTX**	**CAZ**	**CRO**	**CXM**	**IMP**	**CIP**	**LEV**	**CN**	**TOB**	**CHL**
Total (152)	139 (91.45)	56(36.84)	76(50)	81(53.29)	74(48.68)	95(62.50)	61(40.13)	63(41.45)	46(30.26)	63(41.45)	42(27.63)
*E. coli* (67)	62 (92.54)	31 (46.27)	38 (56.72)	39 (58.21)	34 (50.75)	41 (61.19)	32 (47.76)	34 (50.75)	25 (37.31)	30 (44.78)	15 (22.39)
*K. pneumoniae* (34)	32 (94.18)	11 (32.35)	14 (41.18)	16 (47.06)	16 (47.06)	23 (67.68)	14 (41.18)	16 (47.06)	11 (32.35)	17 (50)	11 (32.35)
*C. freundii* (14)	13 (92.86)	3 (21.43)	7 (50)	6 (42.86)	8 (57.14)	11 (78.57)	4 (28.57)	4 (28.57)	2 (14.29)	4 (28.57)	3 (21.43)
*S. liquefaciens* (10)	9 (90)	3 (30)	3 (30)	5 (50)	5 (50)	6 (60)	2 (20)	2 (20)	4 (40)	3 (30)	2 (20)
*S. arizonae* (7)	5 (71.43)	0	2 (28.57)	2 (28.57)	1 (14.29)	2 (28.57)	2 (28.57)	1 (14.29)	1 (14.29)	1 (14.29)	2 (28.57)
*P. mirabilis* (4)	4 (100)	2 (50)	3 (75)	3 (75)	3 (75)	1 (25)	3 (75)	2 (50)	2 (50)	3 (75)	3 (75)
*P. stuartii* (2)	2 (100)	1 (50)	1 (50)	1 (50)	1 (50)	0	0	0	0	2 (100)	2 (100)
*K. ozaenae* (2)	1 (50)	0	1 (50)	2 (100)	1 (50)	0	0	0	0	0	1 (50)
*E. tarda* (2)	2 (100)	1 (50)	2 (100)	2 (100)	1 (50)	2 (100)	1 (50)	1 (50)	0	0	0
* S. rubidae (3)*	3 (100)	2 (66.67)	2 (66.67)	1 (33.33)	1 (33.33)	3 (100)	0 (0)	0	0	1 (33.33)	1 (33.33)
*S. odorifera* (1)	1 (100)	1 (100)	0	1 (100)	1 (100)	1 (100)	1 (100)	1 (100)	0	0	1 (100)
*S. marscesens* (1*)*	1 (100)	0	0	0	0	1 (100)	0	0	0	1 (100)	1 (100)
*S. flexneri (2)*	1 (50)	0	1 (50)	1 (50)	0	2 (100)	0	1 (50)	0	0	0
* S. choleraesius* (1)	1 (100)	1 (100)	1 (100)	1 (100)	1 (100)	1 (100)	1 (100)	1 (100)	1 (100)	1 (100)	0
* S. boydii (1)*	1 (100)	0	0	0	0	0	0	0	0	0	0
* K. oxytoca* (1)	1 (100)	0	1 (100)	1 (100)	1 (100)	1 (100)	1 (100)	0	0	0	0

**AMC**: Amoxicillin-Clavulanic acid; **CTX**: Cefotaxime; **CAZ**: Ceftazidime; **CRO**: Ceftriaxone; **CXM**: Cefixime; **IMP**: Imipenem; **CIP**: Ciprofloxacin; **LEV**: Levofloxacin; **CN**: Gentamicin; **TOB**: Tobramycin; **CHL**: Chloramphenicol

### Antibiotic resistant profiles of ***Enterobacterales*** and ESBL-E 

The antibiotic resistance profiles of *Enterobacterales* species shows a high level of resistance to amoxicillin-clavulanic acid (139/152; 91.45%), imipenem (95/152; 62.5%), ceftriaxone (81/152; 53.29%) and ceftazidime (76/152; 50%) (Table 4). *E. coli* showed the highest resistance rates to amoxicillin-clavulanic acid (92.54%), imipenem (61.19%), ceftriaxone (58.21%), ceftazidime (56.72%), levofloxacin (50.75%), ciprofloxacin (47.76%), chloramphenicol (22,39%) and gentamicin (37.31%). *K. pneumoniae* were mainly resistant to amoxicillin-clavulanic acid (94.18%), imipenem (67.68%) and tobramycin (50%). However, a low resistance level was observed to gentamicin (32.35%) and chloramphenicol (32.35%).

**Table 4:** Distribution of ESBL-Producing *Enterobacterales* and their rate of resistance

Isolates (n)	Prevalence of each species (n (%))	Resistance rate to antibiotics of ESBL PE, n (%)											
	**ESBL positive**	**AMC**	**CTX**	**CAZ**	**CRO**	**CRX**	**IMP**	**CIP**	**LEV**	**CN**	**TOB**	**CHL**	
Total	45 (29.60)	42(93.33)	34(75)	36(80)	36(80)	31(68.89)	28(62.22)	25(55.56)	21(46.67)	11(24.44)	21(46.67)	12(26.67)	
*E. coli* (67)	23 (51.11)	21(91.30)	19(82.61)	20(86.96)	19(82.61)	17(73.61)	13(56.52)	13(56.52)	11(47.83)	6(26.09)	13(56.52)	4(17.39)	
*K. pneumoniae* (34)	8 (17.77)	8(100)	7(87.50)	7(87.50)	7(87.50)	6(75)	5(62.50)	4(50)	4(50)	3(37.50)	5(62.50)	3(37.50)	
*C. freundii* (14)	6 (13.33)	6(100)	3(50)	4(66.67)	4(66.67)	4(66.67)	4(66.67)	3(50)	2(33.33)	0	1(16.67)	2(33.33)	
*P. mirabilis* (4)	1 (02.22)	1(100)	1(100)	1(100)	1(100)	1(100)	0	1(100)	1(100)	1(100)	1(100)	1(100)	
*S. arizonae* (7)	1 (02.22)	0	0	1(100)	1(100)	0	1(100)	1(100)	1(100)	0	0	0	
* S. choleraesius* (1)	1 (02.22)	1(100)	1(100)	1(100)	1(100)	1(100)	1(100)	1(100)	1(100)	1(100)	1(100)	0	
* S. liquefaciens* (10)	1 (02.22)	1(100)	0	0	1(100)	0	1(100)	1(100)	0	0	0	0	
* S. odorifera* (1)	1 (02.22)	1(100)	1(100)	0	1(100)	1(100)	1(100)	1(100)	1(100)	0	0	1(100)	
*S. rubidae* (3)	2 (04.44)	2(100)	2(100)	2(100)	1(50)	1(50)	2(100)	0	0	0	0	1(50)	
*S. boydii* (1)	1 (02.22)	1(100)	0	0	0	0	0	0	0	0	0	0	

**Fig. 2:**
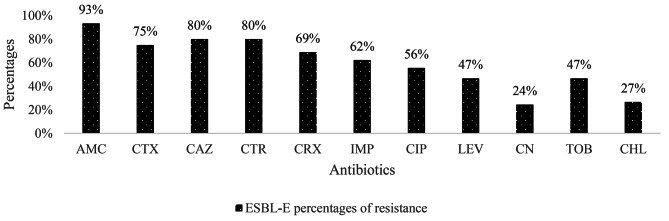
Resistance rate of ESBL-E to antibiotics

Concerning ESBL-E, a significant level of resistance to non β-lactam antibiotics including ciprofloxacin (55.56%), levofloxacin (46.67%) and gentamicin (24.44%) was detected (Table 3; Fig. 2).

### Distribution of multidrug-resistant ***Enterobacterales***

**Fig. 3:**
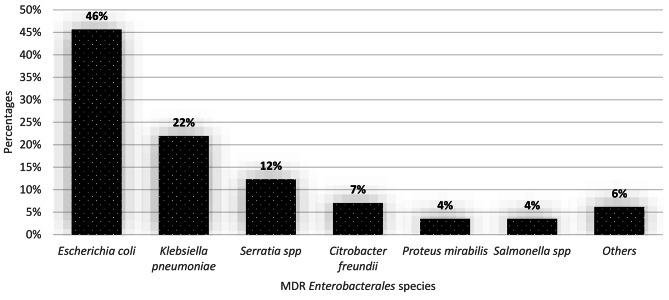
Distribution of MDR*-Enterobacterales* in selected hospitals in Dschang, Cameroon Others*: Edwardiella tarda type 1 (2); K. oxytoca (1); K. Ozaenae (1); P. stuartii (2) and S. flexneri (1)

A total of 114 (75%) isolates were MDR with *E. coli* (n = 52/114; 45.61%) and *K. pneumoniae* (n = 25/114; 21.93%) being the most prevalent MDR species (Table 5; Fig. 3). In addition, all *P. mirabilis* (2.63% n = 4/152) were MDR (Table 5). The vaginal samples were more positive to MDR-*Enterobacterales* (n = 59/114; 51,75%) (Table 4). The majority of MDR-E were resistant to three (n = 39/114; 25.66%) and four (n = 35/114; 23.03%) antibiotic families (Table 5).

**Table 5:** Overall resistance levels of MDR-*Enterobacterales* isolated

Bacterials species (n)	Distribution of multidrug-resistant *Enterobacterales* n (%)				
	**R3**	**R4**	**R5**	**R6**	**Total MDR**
Total (152)	39 (25.66)	35(23.03)	30 (19.74)	10 (6.58)	114 (75)
*E. coli* (67)	16 (23.88)	13 (19.40)	19 (28.36)	4 (5.97)	52 (77.61)
*K. pneumoniae* (34)	7 (20.59)	9 (26.47)	3 (8.82)	6 (17.65)	25 (73.53)
*C. freundii* (14)	1 (7.14)	3 (21.43)	4 (28.57)	0	8 (57.14)
*S. liquefaciens* (10)	7 (70)	2 (20)	0	0	9 (90)
*S. arizonae* (7)	2 (28.57)	1 (14.29)	0	0	3 (42.86)
*P. mirabilis* (4)	1 (25)	1 (25)	2 (50)	0	4 (100)
*S. rubidae* (3)	2 (66.67)	1 (33.33)	0	0	3 (100)
*E. tarda type 1* (2)	1 (50)	1 (50)	0	0	2 (100)
*K. ozaenae* (2)	1 (50)	0	0	0	1 (50)
*P. stuartii* (2)	1 (50)	1 (50)	0	0	2 (100)
*S. flexneri* (2)	0	1 (50)	0	0	1 (50)
*K. oxytoca* (1)	0	1 (100)	0	0	1 (100)
*S. choleraesius* (1)	0	0	1 (100)	0	1 (100)
*S. marscesens* (1)	0	1 (100)	0	0	1 (100)
*S. odorifera* (1)	0	0	1 (100)	0	1 (100)

R3: resistance to 3 different families of antibiotics. R4: resistance to 4 different families of antibiotics. R5: resistance to 5 different families of antibiotics and R6: resistance to 6 different families of antibiotics

**Fig. 4:**
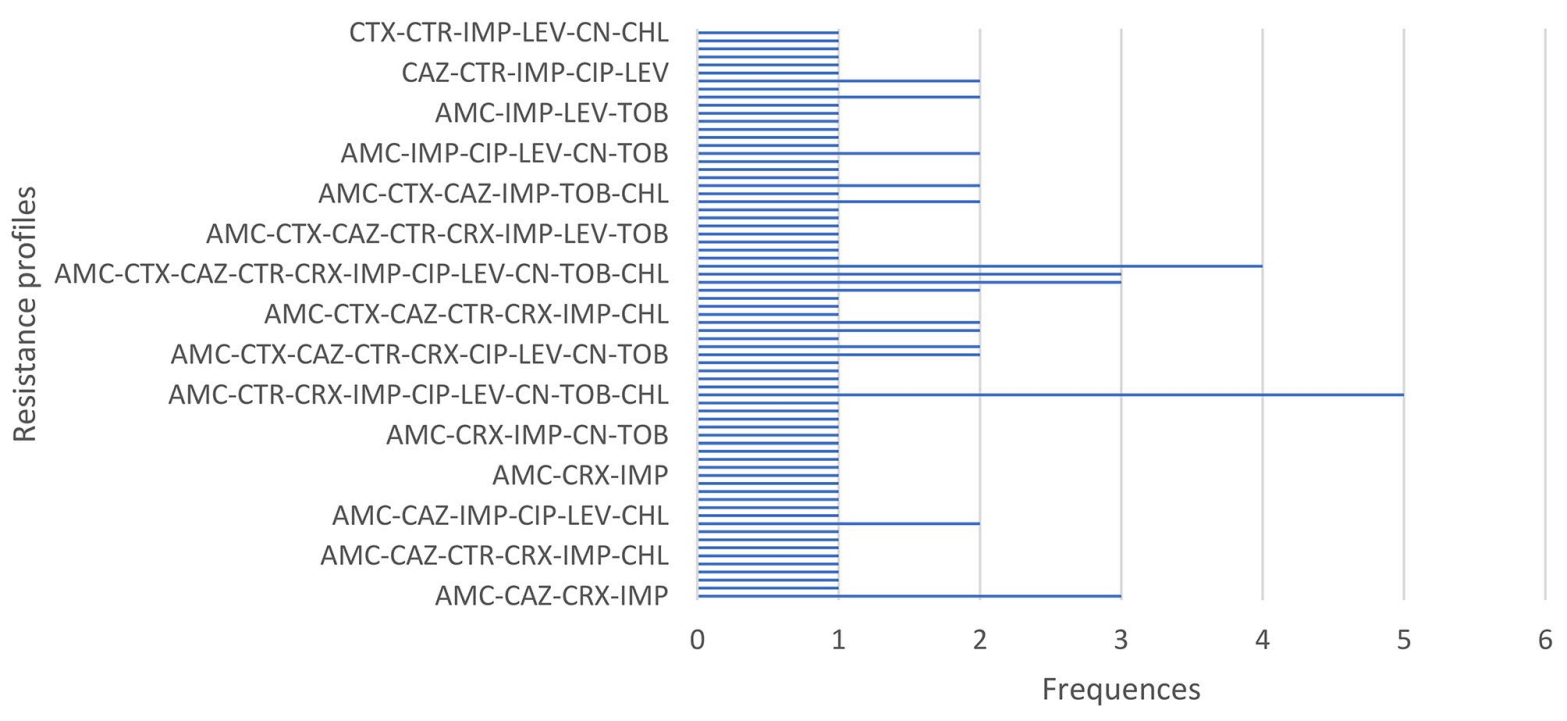
Distribution of MDR*-Enterobacterales* resistance profiles in selected hospitals in Dschang, Cameroon

Among 15 different resistance profiles of MDR-*Enterobacterales*, five isolates (n = 5/114; 4.38%) were resistant to nine antibiotics (**AMC-CTR-CRX-IMP-CIP-LEV-CN-TOB-CHL**) belonging to six different families of antibiotics. Moreover, three isolates (n = 3; 1.97%) were resistant to 11 antibiotics (**AMC-CTX-CAZ-CTR-CRX-IMP-CIP-LEV-CN-TOB-CHL**) from six different families of antibiotics (Fig. 4).

### Characterization of ß-lactamase encoding genes

**Fig. 5:**
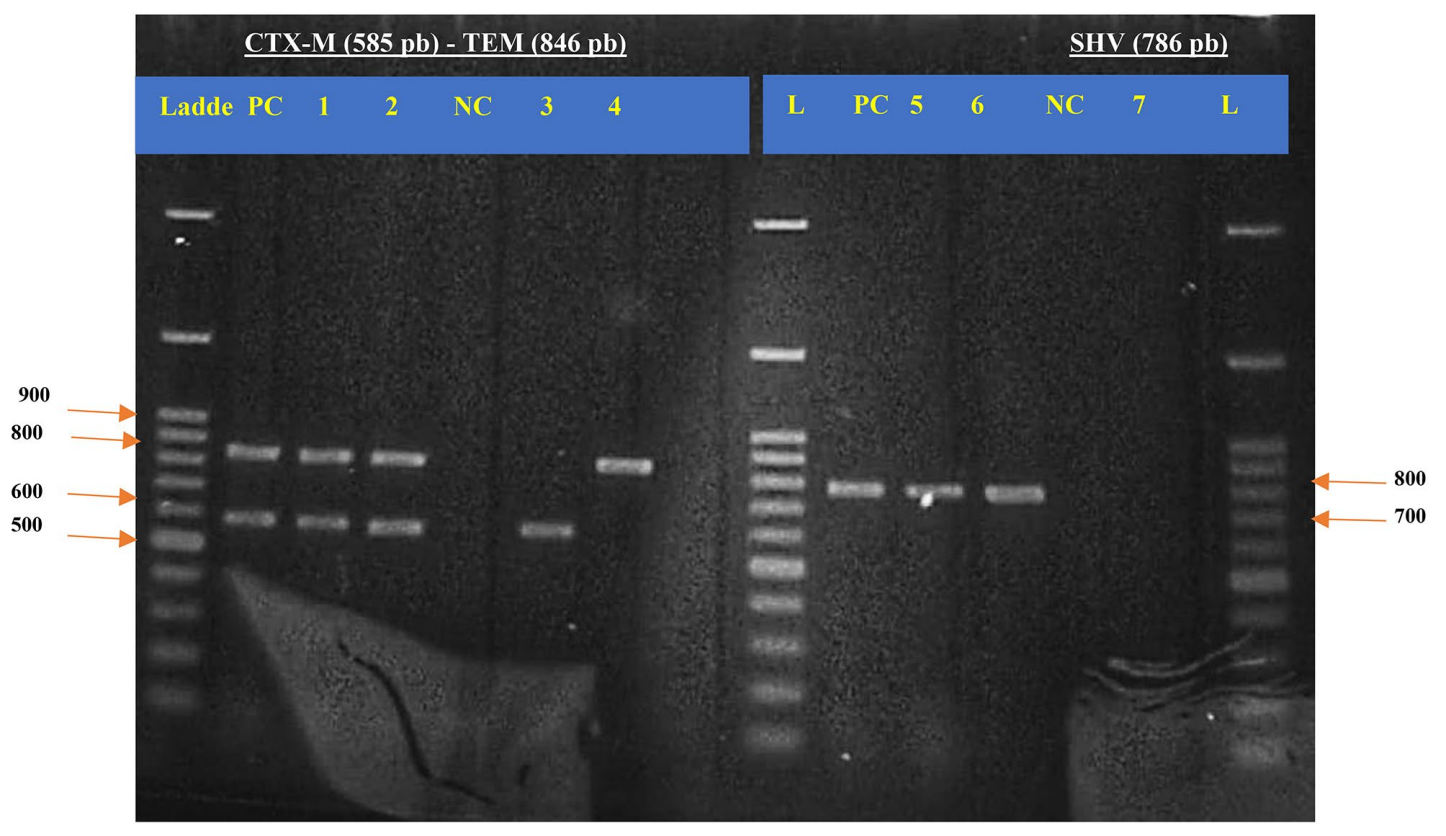
Agarose gel electrophoresis of PCR products (*bla*_CTX-M_, *bla*_TEM_ and *bla*_SHV_) of some ESBL-producing *Enterobacterales* isolated from clinical samples *Ladder: 100 bp DNA ladder; PC: positive control; NC: negative control; 1 & 2: CTX-M positive, TEM positive; 3: CTX-M positive, TEM negative; 4 : CTX-M negative, TEM positive ; 5 & 6 : SHV positive ; 7 : SHV negative*

The results of the molecular characterization shown that *bla*_CTX−M_ (n = 38/45; 84.44%) was the leading ß-lactamase resistance gene, followed by *bla*_TEM_ (n = 45; 73.33%) and *bla*_SHV_ (n = 11; 24.44%) (Fig. 5; Table 6). Interestingly 64.44% (29/45) of ESBL-E isolates carried more than one gene, some isolates carried concomitantly the three genes (n = 10/45; 22.22%). It was reported that 100% (8/8) of ESBL-producing *K. pneumoniae* carried the gene *bla*_CTX−M_ (n = 8/8; 100%) and 75% (6/8) of them carried the three genes (Table 6).

**Table 6:** Distribution of *bla*_CTX-M_. *bla*_TEM_ and *bla*_SHV_ genes in ESBL-E

ESBL-E (n)	Distribution of resistance genes n(%)				Distribution of Co carriages of bla genes n(%)				
	**Single carriage CTX-M or TEM or SHV**	**CTX-M**	**TEM**	**SHV**	**Co-carriage**	**CTX-M + TEM**	**CTX-M + SHV**	**TEM + SHV**	**CTX-M + TEM + SHV**
Total (45)	14 (31.11)	38 (84.44)	33 (73.33)	11 (24.44)	29 (64.44)	28 (62.22)	10 (22.22)	10 (22.22)	10 (22.22)
*E. coli* (23)	9 (39.13)	20 (86.95)	17 (73.91)	2 (08.69)	14 (60.87)	14 (60.86)	2 (08.69)	2 (08.69)	2 (08.69)
*K. pneumoniae* (8)	0	8 (100)	7 (87.5)	7 (87.5)	8 (100)	7 (87.5)	7 (87.5)	6 (75)	6 (75)
*C. freundii* (6)	2 (33.33)	4 (66.66)	6 (100)	1 (16.66)	4 (66.67)	4 (66.66)	1 (16.66)	1 (16.66)	1 (16.66)
*S. rubidae* (2)	0	1 (50)	1 (50)	1 (50)	1 (50)	1 (50)	1 (50)	1 (50)	1 (50)
*P. mirabilis* (1)	0	1 (100)	1 (100)	0	1 (100)	1 (100)	0	0	0
* S. arizonae* (1)	0	1 (100)	1 (100)	0	1 (100)	1 (100)	0	0	0
* S. cholerasius* (1)	1 (100)	1 (100)	0	0	0	0	0	0	0
* S. liquefaciens* (1)	0	0	0	0	0	0	0	0	0
* S. odorifera* (1)	1 (100)	1 (100)	0	0	0	0	0	0	0
* S. boydii* (1)	2 (100)	1 (100)	0	0	0	0	0	0	0

## Discussion

Extended-spectrum β-lactamase-producing *Enterobacterales* remain a public health threat with important societal and economic repercussions as demonstrated [15]. In this study, the prevalence of ESBL-*E* among clinical samples was 29.61% (n = 45/152) in the two highest hospital settings in Dschang district. These results could be explained by the fact that the H1 hospital is in the process of urbanization with the multiplication of street medicine and access of population to self-medication. In addition, this result is higher than those obtained in Central African Republic where the evaluation of increasing prevalence of antimicrobial resistance among ESBL-*Enterobacteriaceae* uropathogens in Bangui shows a prevalence of 19.3% in 2006 [16]. This could also be explained by the fact that they have only worked on urine samples compared to this study where all clinical samples were included. This result is lower than a report observed in Nepal reporting a prevalence (39.13%) of CTX-M β-lactamases producing multi-drug resistant *Escherichia coli* and *Klebsiella pneumoniae* among patients attending Bir Hospital [10]. This difference could be explained by the fact that they have worked on the clinical samples and have selected only MDR-*Enterobacterales* and processed for further ESBL confirmation. Our result is also in contrast with the study conducted in Ghana where the prevalence was (49.3%) and this very high prevalence could be justified by the fact that ESBL-E was an issue already identified and the lack of awareness on misuse and overuse of antibiotics have been previously described [17].

The female (79.84%) was more frequently positive to ESBL-E than male (20.16%). This finding could be explained because endocervical swabs were the most samples collected and the majority of *Enterobacterales* were isolated from this specimen. In addition, these bacteria are commonly observed in the intestinal tract and can colonize the vaginal tract among women given the proximity of the vagina and rectum in women as described by a report conducted by *Bercion et al.* in Central African Republic were 60% of *Enterobacteriaceae* were isolated from woman [16].

Similarly, endocervical swabs (20/45; 44.44%) were the clinical samples most positive to ESBL-E. This finding is in contrast with a study conducted in Sudan in 2020, where most of the ESBL-E isolates were detected from urine samples (44%, n = 75) and wound swabs (44%, n = 75) respectively [18]. This could be explained by the fact that female was most represented in the study and observed in this region. Moreover, the lack of water, poor hygienic conditions combined with consumption of counterfeit antibiotics, poor education level could contribute to the self-contamination [9].

This study reveals the predominance of *E. coli* (n = 67; 44.08%), *K. pneumoniae* (n = 34; 24.34%) and *C. freundii* (n = 14; 9.21%). These results are similar to those reported in Uganda, during a study carried out in Mulango hospital concerning clinical samples in which they identify *E. coli* as the most organism (53.9%), followed by *K. pneumoniae* (28.7%) [8].

This is in agreement with a study conducted in Ethiopia by Teklu et al. in 2017, where *Enterobacterales* especially *E. coli* (n = 228; 53.5%) and *K. pneumoniae* (n = 103; 24.1%) were the most bacteria isolated among clinical samples [6]. In addition, *E. coli* (n = 23; 34.33%) and *K. pneumoniae* (n = 8; 23.53%) were the leading ESBL producers. Our findings are similar to several studies investigated worldwide especially in Africa countries like Burkina Faso, where a study shown that ESBL-E were mostly represented among clinical samples with *E. coli* (67.5%) and *K. pneumoniae* (26%) [19], in Ethiopia where *E. coli* (52.2%) and *K. pneumoniae* (78.6%) were the most detected ESBL-E in clinical samples collected from hospitals in Addis Ababa [6], and in study reported in Uganda where *K. pneumonia* had the highest rate of ESBL producers (72.7%) among clinical samples in Mulago hospital after a cross-sectional study in 2014 [8].

ESBL-E showed the high resistance rates of more than 60% to all ß-lactams family including penicillin, cephalosporins and carbapenems. This result is similar to those obtained in Nigeria in 2016, where high resistance rate was observed among ESBL-E isolates with the high resistance profile with ceftriaxone (92.3%), aztreonam (96.8%), cefpodoxime (96.3%), cefotaxime (98.8%) and trimethoprim/sulfamethoxazole (90%) [20]. This high resistance level to ß-lactams family of ESBL-E has been already described by Teklu et al. in Ethiopia with ceftazidime (100%) and ceftriaxone (100%) [6].This could be explained by the extensive and unregulated use of β-lactam antibiotics as a first-line medication to treat infectious diseases in Africa [9, 10]. Numerous reports have already demonstrated that in African countries, hospitalized patients received numerous antibiotics for the therapeutic purpose without antibiotic susceptibility testing, which contribute to the selective pressure on the microbiome and increase antimicrobial resistance [21]. High level of MDR-*E. coli* and MDR-*K. pneumoniae* have been observed and this could be explained by the excessive and inappropriate use of antibiotics in Dschang, where antibiotics are easily accessible over the counter without a prescription [22].

ESBL-*E. coli* and ESBL-*K. pneumoniae* are commonly responsible of hospital-and community-acquired infections [18]. The *bla*_CTX−M_ and *bla*_TEM_ were the most frequent ß-lactamase genes detected among ESBL-*E. coli* and ESBL-*K. pneumoniae* with prevalence ranging from 74 to 100%. These findings are in agreement with those obtained in Nepal where the prevalence of *bla*_CTX−M_ among clinical ESBL-*E. coli* and ESBL-*K. pneumoniae* were 93.81% and 78.94% isolated from clinical samples respectively [10]. This finding also agreed with a study conducted in Egypt where *bla*_CTX−M_ was the most common ESBL genes among ESBL-producing *E. coli* with a prevalence of 83.3% [21]. These results could be explained by the powerful ability of this gene to hydrolyze numerous β-lactam antibiotics including ceſtazidime, cefotaxime and aztreonam, which probably offers a selective advantage when they are overuse or misuse concomitantly. Moreover, it is plausible because the third generation of cephalosporins including ceftriaxone and cefixime are extensively used as a first line treatment among hospitalized and community patients having vaginal, urinary tract and bloodstream infections in the H1 and H2 hospitals, this could contribute to the expansion of *bla*_CTX−M_ in the region [23]. However, the relatively low prevalence of *bla*_SHV_ has already been reported in Africa countries especially in Chad where there had no *bla*_SHV_ gene among clinical samples [9]. This result agrees with the results obtained in numerous studies conducted during the last ten years in LMIC countries and confirm that *bla*_SHV_ is relatively infrequent in Africa [2].

### Limits

The limitations of our study are the unable detect the genes responsible for carbapenem resistance, or to detect other resistance gene coding for β-lactam resistance. In addition, this study was limited to two health facilities in the Dschang district, it would be interesting to extend the study to the whole of Cameroon to obtain more relevant results.

## Conclusion

This study reveals the high burden of MDR-E and ESBL-E among clinical samples in the regional hospital in Dschang with the most common species being *E. coli* and *K. pneumoniae*. It confirmed the high occurrence of *bla*_CTX−M_ and *bla*_TEM_ among ESBL-E. The study suggests that implementing antimicrobial stewardship program and real-time surveillance of antimicrobial resistance are needed in the Western region of Cameroon. Moreover, the implementation of infection prevention and control measures (IPC) is essential to curb the dissemination of these bacteria from community to hospital settings. It will be also important to reduce the burden and subsequent morbidity and mortality rates associated with antimicrobial resistance in the country by implementing national action plan to fight against antimicrobial resistance at the local levels.

## Data Availability

The data are available upon request in accordance with confidentiality and privacy regulations from the corresponding author.

## Abbreviations

MDR: multidrug resistance
ESBL: Extended Spectrum β-lactamase
ESBL-E: Extended Spectrum β-lactamase producing-*Enterobacterales*
